# Hereditary motor and sensory neuropathy with SOD1-mutant: A case report

**DOI:** 10.1097/MD.0000000000031378

**Published:** 2022-10-28

**Authors:** Zhong Luo, Linhai Zhang, Juan Yang, Haiqing Zhang, Tao Liang

**Affiliations:** a Department of Neurology, Affiliated Hospital of Zunyi Medical University, Dalian road. Zunyi, China.

**Keywords:** hereditary motor and sensory neuropathy, pedigree, *SOD*1-mutant

## Abstract

Hereditary motor-sensory peripheral neuropathy, or Charot-Marie-Tooth (CMT) Charcot-Marie-Tooth disease is an inherited peripheral neuropathy characterized by progressive limb weakness and muscle atrophy. As the disease progresses, sensory and autonomic involvement may occur. We report a case of CMT associated with *SOD1* gene mutation, in order to provide new ideas for clinical disease diagnosis.

**Patient concerns:**

A 50-years-old female patient was admitted to the hospital with “progressive weakness of the right lower extremity for 5 years, aggravating, and weakness of the left lower extremity for 4 months”.

**Diagnosis:**

The patient was diagnosed CMT.

**Intervention:**

Nerve nutrition and rehabilitation therapy were given, but the patient’s condition still did not improve significantly.

**Outcomes:**

The improvement of symptoms was not obvious.

**Lessons:**

The clinical manifestations and electromyography results of this patient are consistent with the characteristics of CMT. The peripheral nerve-related hereditary gene test found mutation in *SOD1*. It is possible that this mutation is linked to CMT. The disease is a neurodegenerative disease, that may be slowed by physical therapy and rehabilitation, but could not be healed.

## 1. Introduction

Hereditary motor-sensory neuropathy (HMSN), also known as peroneal muscle atrophy, is a common hereditary peripheral neuropathy that primarily manifests as progressive limb muscle weakness and muscle atrophy.^[1]^ As the disease progresses, symptoms of sensory and vegetative involvement may occur.^[1]^ According to clinical and electrophysiological characteristics, it can be categorized as demyelinating type, axonal type or intermediate type. At present, the most common genetic pathogenic loci include PMP22, GJB1, MFN2 and MPZ, and these account for more than 90% of all subtypes of the disease.^[2]^ In recent years, with the development and application of gene sequencing technology, more than 90 pathogenic genes of other mutation sites and families have been discovered and reported.^[3]^ However, to date, cases of *SOD*1 gene mutations in HMSN patients and families are rarely reported. This paper reports the clinical characteristics, diagnosis and treatment of a family of HMSN patients with *SOD*1 gene mutations admitted to our hospital, hoping to provide some new traces and details that may help with the diagnosis and treatment methods of this disease.

## 2. Case presentation

The subject was a 50-years-old woman who was admitted to the hospital due to “progressive weakness of the right lower limb for 5 years, aggravated and left lower limb weakness for 4 months”. The patient presented with a 5-years history of right lower limb weakness and right foot pain without obvious incentives, right lower limb numbness, paraesthesia or muscle tremor. Electromyography (EMG) was conducted, and the results suggested abnormal somatosensory evoked potential on the left side of the posterior tibial nerve and common peroneal nerve and moderately abnormal somatosensory evoked potential on the right side of the posterior tibial nerve and common peroneal nerve. The patient and her family did not attach importance to the symptom, so she went without further treatment. In the last 2 years, the weakness of the right lower limb progressively worsened, that she had to use crutches, and there was muscle atrophy in the right lower limb, mainly gastrocnemius atrophy. The patient went to Chongqing Southwest Hospital and finished an EMG examination. The results showed that the left common peroneal nerve did not elicit motor conduction velocity. The motor conduction velocity of right common peroneal nerve and bilateral tibial nerves slowed down, the amplitude decreased, and the incubation period was prolonged. She was given nerve nutrition treatment, and her conditions improved slightly after treatment. Four months ago, the patient had left lower limb weakness and fell without obvious cause. Then, the weakness in her left lower limbs progressively worsened that she could not walk, but there was no dizziness or headache, she was admitted to our hospital with “lower motor neuron syndrome”. At the onset of the illness, the mental state, diet, sleep, stool and urination were normal, and there was no significant increase or decrease in weight.

The patient was previously healthy and denied a history of any particular illness. The family history was obtained. The patient’s father had a similar history. He developed the disease at the age of 50 and died at the age of 70 (details are not available). The physical examination at admission revealed that the vital signs were stable. The patient was conscious and was brought into the ward with a wheelchair. No abnormalities were found in the physical examination. The nervous system examination revealed that the patient’s speech was not clear, and no abnormalities were found in the examination of advanced cognitive function. The muscle strength of the distal right upper extremity was grade 4, grip strength was decreased, muscle strength of the left upper extremity was grade 4+, muscle strength of the right lower extremity was grade 1, muscle strength of the left lower extremity was grade 2–, muscle tension of the extremities was weakened, muscle atrophy of both of the distal lower extremities was observed, no limb paraesthesia was observed, right biceps and triceps reflexes were not elicited, the tendon reflexes of both lower extremities were not elicited, and the Babinski sign was positive.

**Figure 1.**
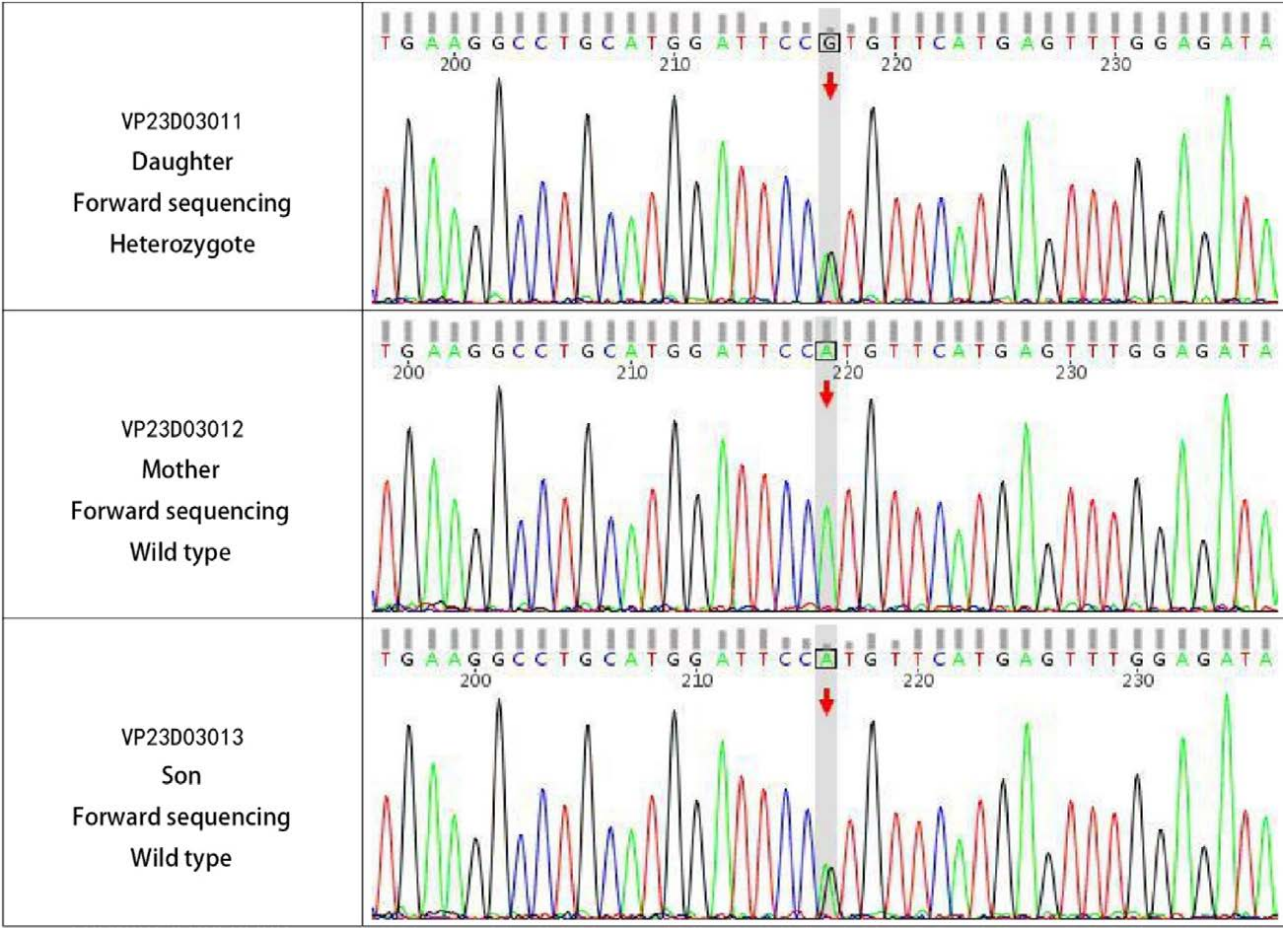
Gene exon sequencing test report of proband and pedigree.

An auxiliary examination was conducted (February 28, 2019 Chongqing Southwest Hospital). The EMG revealed that the left common peroneal nerve motor conduction potential was not elicited, the right common peroneal nerve and bilateral tibial nerve motor conduction velocity slowed down, the wave amplitude decreased, the latency prolonged, and the H wave conduction velocity of the left tibial nerve slowed down. A thoracolumbar MRI was performed (July 09, 2015 our hospital) and revealed degenerative changes of the lumbar spine. The ECG on admission was normal. Blood routine examination, 3 items of anemia, thyroid function, liver and kidney function, and antinuclear antibody spectrum showed no abnormalities. The EMG of sensory conduction velocity in the extremities revealed multiple peripheral nerve lesions. The ganglioside antibody spectrum (blood, cerebrospinal fluid), routine cerebrospinal fluid and cerebrospinal fluid biochemistry showed no abnormalities. The results of genetic testing of cerebrospinal fluid for hereditary peripheral neuropathy showed that both the proband and her daughter share the mutation in gene *SOD*1 CHR21:33036170 Exon2 NM_000454.5: C.140A > G (P.HiS47Arg) (Fig. 1). The proband’s spouse, mother and son were normal. The patient was admitted to the hospital with a diagnosis of hereditary motor and sensory neuropathy. After admission, she was actively given neurotrophic nerve and symptomatic treatment, but the improvement of symptoms was not obvious.

## 3. Discussion and conclusions

HMSN, also known as Charcot-Marie-Tooth neuropathy (CMT neuropathy), is a group of heterogeneous motor and sensory genetic neuropathies that was first reported by Charcot, Marie and Tooth in 1886.^[4]^ The pathology of HMSN is dominated by reduced nerve conduction velocity, hypertrophic demyelination and axonal lesions. The clinical symptoms primarily manifest as progressive limb muscle weakness, muscular atrophy, difficulty in walking and foot deformity, accompanied by obvious sensory and vegetative nerve damage in the later stage.^[5]^ At present, there is no effective cure for the disease, and the disease can only be controlled by physical therapy and rehabilitation therapy. Therefore, early diagnosis can positively and correctly guide HMSN patients to change their lifestyle to minimize neurological damage as much as possible, thus delaying or preventing the disability rate of the disease^[6]^.

HMSN is a genetic disease with diverse inheritance methods. The genetic modes of HMSN include autosomal dominant inheritance, autosomal recessive inheritance, X-linked dominant inheritance and recessive inheritance. To date, approximately 90 gene mutations have been identified to be related to the incidence of this disease, and the overall prevalence is approximately 1/2500.^[7]^ It can be categorized into different subtypes according to genetic loci and pathogenic genes. CMT1A is caused by a *PMP22* gene mutation and is the most common subtype, accounting for more than 70% of all subtypes. Other common pathogenic loci include *GJB1*, *MFN2*, and *MPZ*.^[8]^ With the widespread application of gene sequencing technology, some familial and sporadic HMSN cases caused by rare site mutations have been reported in recent years.^[9]^

Studies have shown that HMSN is often accompanied by acute, subacute or chronic inflammatory responses, which can lead to an increase in oxidative stress and reactive oxygen species. Therefore, oxidative stress and neuroinflammation may be involved in the pathogenesis of HMSN. However, antioxidants and/or anti-inflammatory therapy can inhibit the hyperoxidative state and neuroinflammatory response. Therefore, some researchers have suggested that antioxidant and/or anti-inflammatory therapy may be beneficial to HMSN.

*SOD*1 is the most important antioxidant enzyme in the SOD family and plays a key role in the endogenous defence system of antioxidants. *SOD*1 is mainly distributed in the cytoplasm, nucleus, peroxisomes and mitochondrial membrane space of eukaryotic cells and the periplasmic space of bacteria.^[10]^ In 1993, Rosen et al found that a *SOD*1 gene point mutation was associated with familial amyotrophic lateral sclerosis (ALS).^[11]^ Subsequent studies showed that the *SOD*1 gene is a common pathogenic mutation site of ALS, accounting for approximately 10-20% of familial *ALS* gene mutations.^[12,13]^ With further research, *SOD*1 gene mutations are associated with various neurodegenerative diseases, such as Alzheimer’s disease and Parkinson’s disease.^[14,15]^ Long-term follow-up of a case of ALS with a *SOD*1 gene mutation found that with the prolongation of the disease course, the patient’s symptoms of medullobulbar and respiratory dysfunction disappeared completely. However, the other clinical symptoms and neuroelectrophysiological evidence supported the diagnosis of HMSN axonal type. Therefore, researchers disagree on the diagnosis of ALS, believing that HMSN may be combined with a specific unknown mechanism.^[3]^ A *SOD*1 gene mutation was found in a CMT family through gene sequencing, and it was speculated that *SOD*1 gene mutations might be the pathogenic site of HMSN.^[16,17]^ Affected individuals in another large CMT2 family carry a pathogenic variant of *SOD*1.^[18]^ However, cases of HMSN caused by *SOD*1 gene mutations have not been seen reported.

In this case, progressive lower extremity weakness with muscular atrophy started with right lower extremity weakness and gradually spread to the left lower extremity. The movement was significantly limited and accompanied by muscle atrophy, particularly gastrocnemius atrophy. The father in the family has a similar history. The physical examination revealed that no positive signs were found in higher nerve function, and both lower limbs showed signs of lower motor neuron damage accompanied by pyramidal bundle signs. These findings indicate that the upper motor neuron was affected. Multiple EMG examinations indicated that the motor conduction velocity of the bilateral common peroneal nerve and bilateral tibial nerve slowed down, wave amplitude decreased, latency prolonged, and H wave conduction velocity of the left transverse nerve slowed down. No fibrillation potential or positive sharp waves were observed. Routine biochemistry and ganglioside antibodies of cerebrospinal fluid were negative. The above physical and clinical examinations, in conjunction with the clinical symptoms the patient had, supported a diagnosis of peripheral neuropathy. Genetic peripheral neuropathy test results indicated that both the proband and her daughter had a mutated gene: *SOD*1 CHR21:33036170 Exon2 NM_000454.5: C.140A > G (P. His47Arg). The mutation of the *SOD*1 gene in this family is a common mutation site of ALS, which requires careful identification. However, the patient’s multiple EMG changes were not consistent with the changes in ALS. Combined with previous reports that *SOD*1 gene mutations may be associated with CMT, the pathogenic site of the CMT gene mutation is mainly considered. The final diagnosis was hereditary motor and sensory neuropathy. The disease is a neurodegenerative disease, and its progression can only be delayed by physical and rehabilitation treatment. Therefore, the symptoms do not improve significantly after active neurotrophic treatment. We hope that this case report allows clinicians to raise awareness of the fact that *SOD*1 can cause peripheral neuropathy damage.

## Author contributions

**Conceptualization:** Zhong Luo, Linhai Zhang.

**Methodology:** Haiqing Zhang.

**Data curation:** Zhong Luo.

**Investigation:** Haiqing Zhang.

**Validation:** Juan Yang.

**Resources:** Zhong Luo.

**Project administration:** Juan Yang, Tao Liang.

**Writing – original draft:** Zhong Luo, Linhai Zhang.

**Writing – review & editing:** Tao Liang.
